# Mesenchymal stem cells rejuvenate cardiac muscle through regulating macrophage polarization

**DOI:** 10.18632/aging.102009

**Published:** 2019-06-18

**Authors:** Busheng Zhang, Naishi Zhao, Jing Zhang, Yu Liu, Dan Zhu, Ye Kong

**Affiliations:** 1 Department of Cardiac Surgery, Shanghai Chest Hospital, Shanghai Jiao Tong University, Shanghai 200030, China; 2 Department of Cardiac Surgery, The Second Hospital of Hebei Medical University, Shijiazhuang 050000, China

**Keywords:** mesenchymal stem cells (MSCs), myocardial infarction (MI), macrophage polarization

## Abstract

We have shown that the effects of transplantation of CD146+ mesenchymal stem cells (MSCs) on myocardial regeneration after myocardial infarction (MI) exceeds the effects of transplantation of MSCs, likely resulting from reduction of aging-associated cellular reactive oxygen species in injured cardiac muscle cells (CMCs). Since the role of macrophages in the MSC-mediated recovery of heart function after MI remains unclear, this question was thus addressed in the current study. We found that transplantation of MSCs did not alter the total number of the macrophages in the injured heart, but induced their polarization towards a M2-phenotype. Moreover, administration of tumor necrosis factor alpha (TNFα) into MSC-transplanted mice, which prevented M2-polarization of macrophages, abolished the effects of MSCs on recovery of heart function and on the reduction of infarcted cardiac tissue. Thus, our data suggest that MSCs may rejuvenate CMCs after ischemic injury at least partially through induction of M2-polarization of macrophages.

## INTRODUCTION

Myocardial infarction (MI) is a major cause of coronary heart disease (CHD), and often causes ischemic cardiomyopathy and heart failure [1]. Cardiomyocytes are potently injured in the disease and appear to be the most critical cell type that requires effective regeneration or recovery from severe dysfunction to result in a successful therapy [2].

More and more studies have shown that stem cells can play an important role in tissue repair and anti-inflammation. In particular, mesenchymal stem cells (MSCs) have shown anti-inflammatory and immunological functions. Indeed, MSCs have also been shown to have the potential to enhance the recovery and regeneration of the infarcted myocardium [3–6]. The current belief on the role of MSCs in myocardial regeneration is their synthesis and secretion of cytokines and other trophic growth factors to signal to the injured myocardial cells [7], which may also involve anti-aging effects [8–10].

MSCs express specific surface markers, CD105, Sca-1, and CD90, and do not express CD34, CD45, and HLA-DR [11–13]. Moreover, MSCs have multipotent differential capabilities of osteocytes, adipocytes and chondrocytes [14]. These properties are used to characterize MSCs. CD146 in a marker that expresses in capillary pericytes [15]. We have recently shown that the effects of transplantation of CD146+ MSCs on myocardial regeneration after MI exceeds the effects of transplantation of MSCs, likely resulting from reduction of aging-associated cellular reactive oxygen species in injured cardiac muscle cells (CMCs) [16].

Many effects of MSCs on tissue repair and cell regeneration are conducted through their crosstalk with macrophages [17–19]. It is traditionally thought that Macrophage are deemed to be white blood cells with a major functionality of swallowing and ingesting wastes, dying or dead cells, and impurities [20–23]. Nevertheless, recently studies have shown that macrophages have much more functions other than phagocytosis. Therefore, a more complicated classification of macrophages has been applied, in which 2 subtypes of macrophages are distinguished by two phenotypes. One was named as “M1” macrophages, while the other alternatively polarized one was named as “M2” macrophages, which function in regulation of humoral immunity and promotion of tissue repair [20–23]. CD206, CD163, arginase and CD301 are expressed by M2 macrophages, high levels of CD86, nitric oxide synthase (iNOS), reactive oxygen species (ROS) and tumor necrosis factor alpha (TNFα) are expressed by the M1 macrophages [20–23]. Since the role of macrophages in the MSC-mediated recovery of heart function after MI remains unclear, this question was thus addressed in the current study.

We found that transplantation of MSCs did not alter the total number of the macrophages in the injured heart, but induced their polarization towards a M2-phenotype. Moreover, administration of TNFα into MSC-transplanted mice, which prevented M2-polarization of macrophages, abolished the effects of MSCs on recovery of heart function and on the reduction of infarcted cardiac tissue.

## RESULTS

### Confirmation of MSC properties

**Figure 1:**
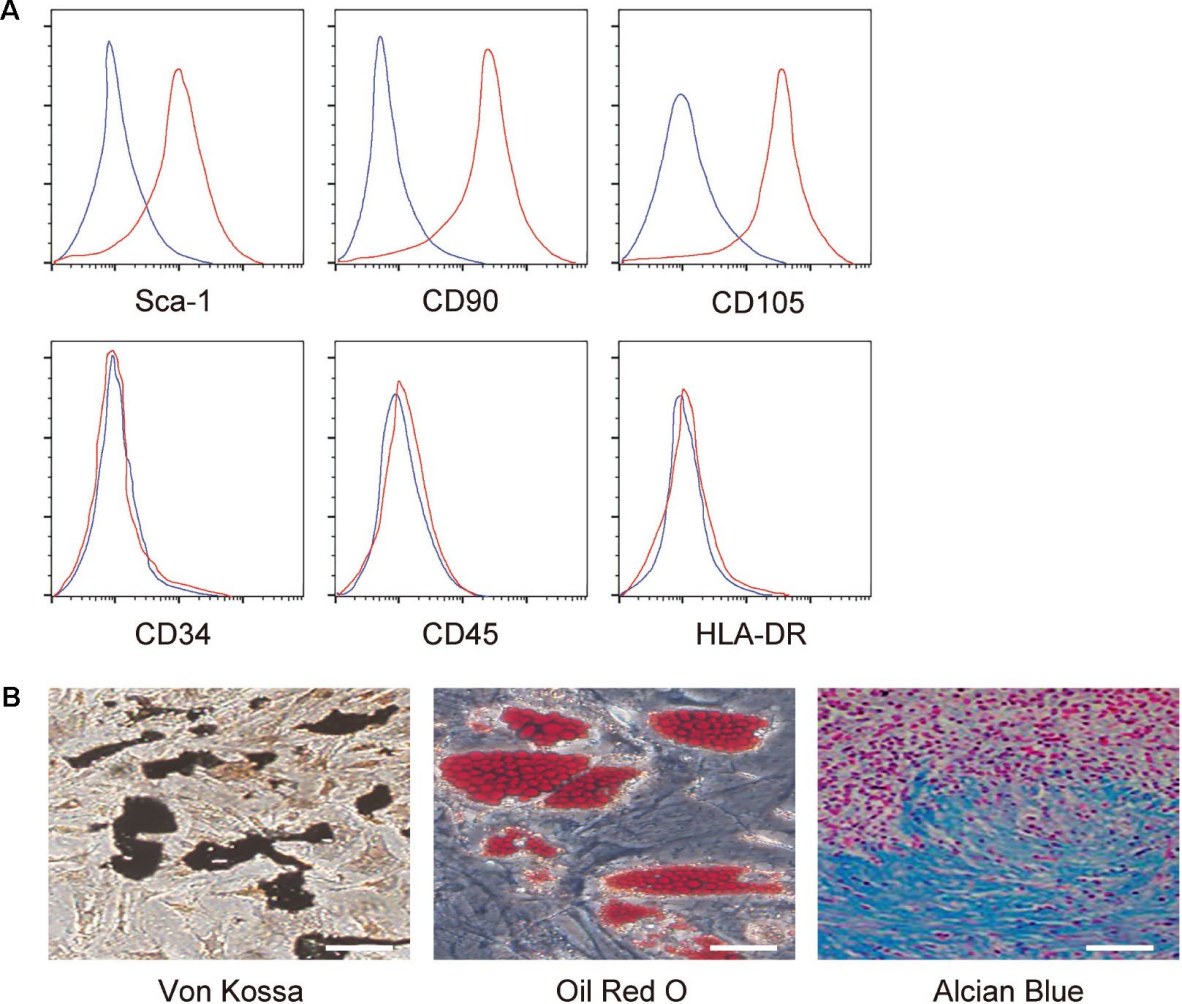
**Confirmation of MSC properties.** (**A**) Mouse MSC surface markers (Sca-1, CD90, CD105, CD34, CD45 and HLA-DR) were examined by flow cytometry. Y-axis is the number of the cells, and the large scale is 10^5^. X-axis is the level of the examined gene, and the large scale is a relative density value. (**B**) Differentiation assay for MSCs into osteocytes followed by Von kossa staining (left), into adipocytes followed by Oil red O staining (middle), and into chondrocytes followed by alcian blue staining (right). N=5. Scale bars are 50 μm.

MSCs were isolated from mice and the MSC properties were confirmed sequentially by FAC-analysis on the expression of surface markers, including expression of Sca-1, CD90 and CD105, but null expression of CD34, CD45 and HLA-DR (Figure 1A), and by full potential of differentiation into osteocytes, adipocytes or chondrocytes in the corresponding differentiation media (Figure 1B).

### Transplantation of MSCs induces M2-polarization of macrophages

**Figure 2:**
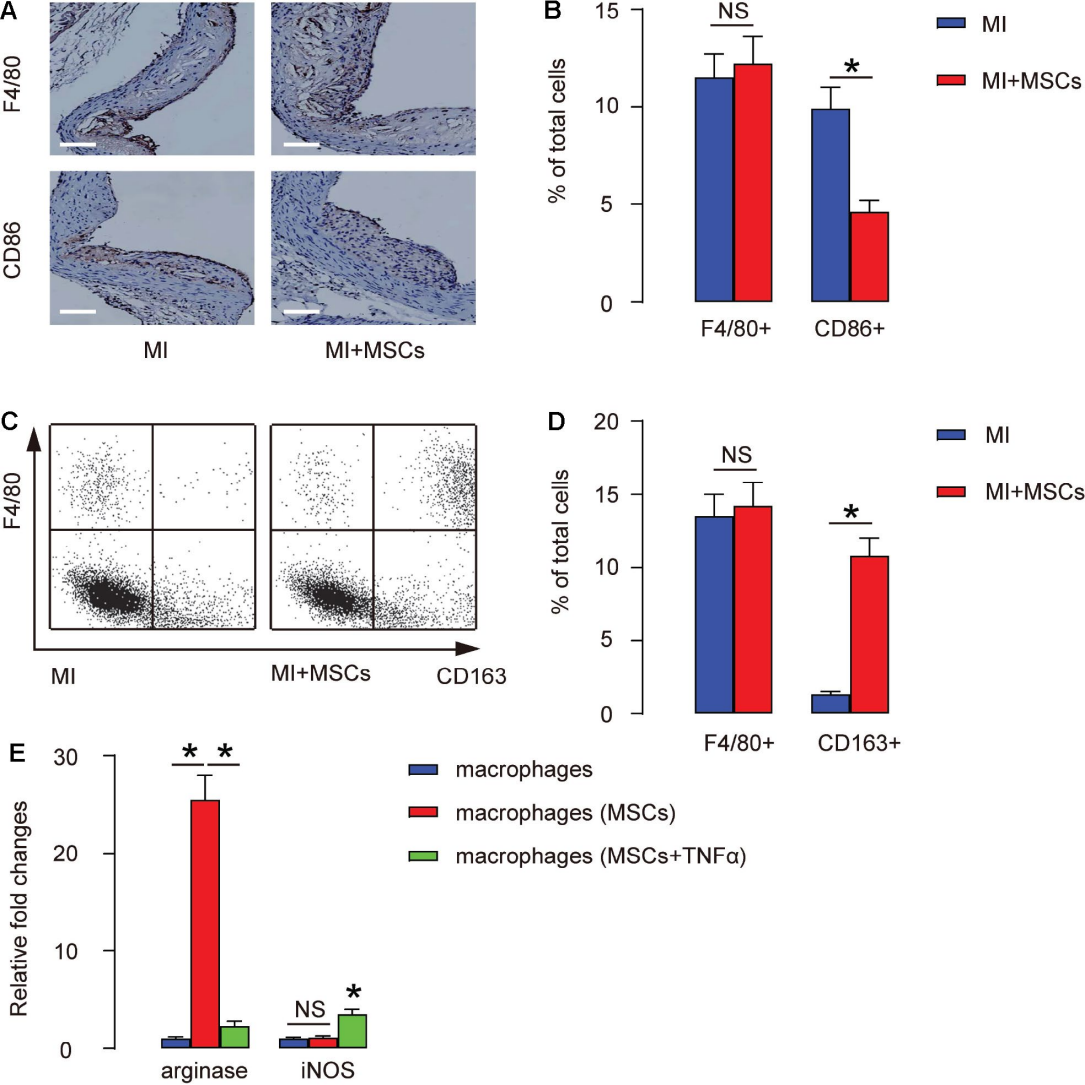
**Transplantation of MSCs induces M2-polarization of macrophages**. (**A**–**B**) MSCs were transplanted into MI mouse model for 4 weeks, followed by immunostaining for F4/80, a pan-macrophage marker, or CD86, a M1-specific macrophage marker, in the injured heart, shown by representative images (**A**), and by quantification (**B**). (**C**–**D**) The injured heart at 4 weeks after transplantation was digested, and the dissociated cells were analyzed by F4/80 and CD163, a M2-specific macrophage marker, shown by representative flow charts (**C**), and by quantification (**D**). Y-axis is F4/80 staining, and the X-axis is CD163 staining. (**E**) ELISA for arginase and iNOS in cultured macrophages, with/without presence of MSCs, and with/without presence of TNF-α. *p<0.05. NS: non-significant. N=5. Scale bars are 100 μm.

MSCs were transplanted into MI mouse model as described [16]. We did immunostaining for F4/80, a pan- macrophage marker, or CD86, a M1-specific macrophage marker, in the injured heart at 4 weeks after transplantation. We found that the total number of macrophages (by ratio of F4/80+ to the total cells) in the MI-heart did not alter by MSC transplantation, shown by representative images (Figure 2A), and by quantification (Figure 2B). However, the total number of M1 macrophages (by ratio of CD86+ to the total cells) in the MI-heart significantly decreased by MSC transplantation, shown by representative images (Figure 2A), and by quantification (Figure 2B). These data indicate that M2 macrophages may increase by MSC transplantation. To prove it, we digested the injured heart at 4 weeks after transplantation, and analyzed the dissociated cells by F4/80 and CD163, a M2-specific macrophage marker. Again, by FACS, we found that the total number of macrophages (by ratio of F4/80+ to the total cells) in the MI-heart did not alter by MSC transplantation, shown by representative flow charts (Figure 2C), and by quantification (Figure 2D). However, the total number of M2 macrophages (by ratio of CD163+ to the total cells) in the MI-heart significantly increased by MSC transplantation, shown by representative flow charts (Figure 2C), and by quantification (Figure 2D). Together, these data suggest that transplantation of MSCs does not alter the total number of the macrophages in the injured heart, but induces their polarization towards a M2-phenotype. In order to prove it, MSCs and bone marrow derived macrophages were cultured in a transwell with and without presence of TNF-α, a cytokine that enhances M1- but prevents M2- differentiation of macrophages. We found that MSCs significantly increased arginase but did not change iNOS levels in macrophages (Figure 2E), while the effects of MSCs on arginase were significantly attenuated at presence of TNF-α (Figure 2E).

### MSC-induced M2-polarization of macrophages is essential for protection of heart function in MI-mice

**Figure 3:**
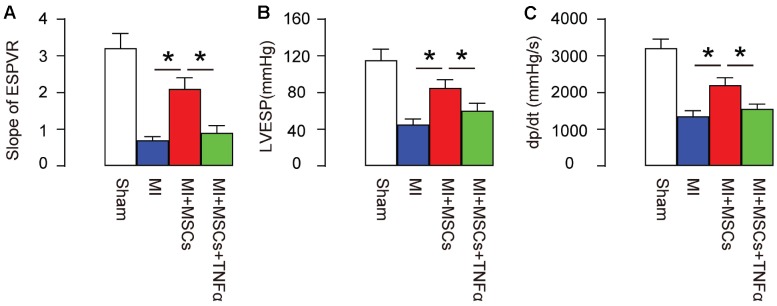
**MSC-induced M2-polarization of macrophages is essential for protection of heart function in MI-mice**. In order to evaluate the function of MSC-induced M2-polarization of macrophages in MI-mice, we set up 4 groups of mice. Group 1, mice received sham surgery and injection of saline (Sham). Group 2, mice received MI surgery and injection of saline (MI). Group 3, mice received MI surgery and injection of MSCs (MI+MSCs). Group 4, mice received MI surgery, injection of MSCs and injection of TNF-α, which helps to maintain a M1-phenotype of macrophages (MI+MSCs+ TNF-α). Four weeks after MI/MSCs/TNF-α treatment, the mouse heart function was assessed using ventricular catheterization. (**A**) End systolic pressure-volume relationship (ESPVR) (**B**) left ventricular end systolic pressure (LVESP) (**C**) Positive maximal pressure derivative (+dP/dt). *p<0.05. N=5.

In order to evaluate the function of MSC-induced M2-polarization of macrophages in MI-mice, we set up 4 groups of mice. Group 1, mice received sham surgery and injection of saline (Sham). Group 2, mice received MI surgery and injection of saline (MI). Group 3, mice received MI surgery and injection of MSCs (MI+MSCs). Group 4, mice received MI surgery, injection of MSCs and injection of TNF-α, which helps to maintain a M1-phenotype of macrophages (MI+MSCs+ TNF-α). Four weeks after MI/MSCs/TNF-α treatment, the mouse heart function was assessed using ventricular catheterization. We found that end systolic pressure-volume relationship (ESPVR) was significantly impaired in MI, compared to Sham. Transplantation of MSCs significantly improved ESPVR, which was significantly attenuated by TNF-α (Figure 3A). Moreover, left ventricular end systolic pressure (LVESP) was significantly impaired in MI, compared to Sham. Transplantation of MSCs significantly improved LVESP, which was significantly attenuated by TNF-α (Figure 3B). Furthermore, positive maximal pressure derivative (+dP/dt) was significantly impaired in MI, compared to Sham. Transplantation of MSCs significantly improved dP/dt, which was also significantly attenuated by TNF-α (Figure 3C). Hence, MSC-induced M2-polarization of macrophages appears to be essential for protection of heart function in MI-mice.

### MSC-induced M2-polarization of macrophages is essential for reducing infarction area in MI-mice

**Figure 4:**
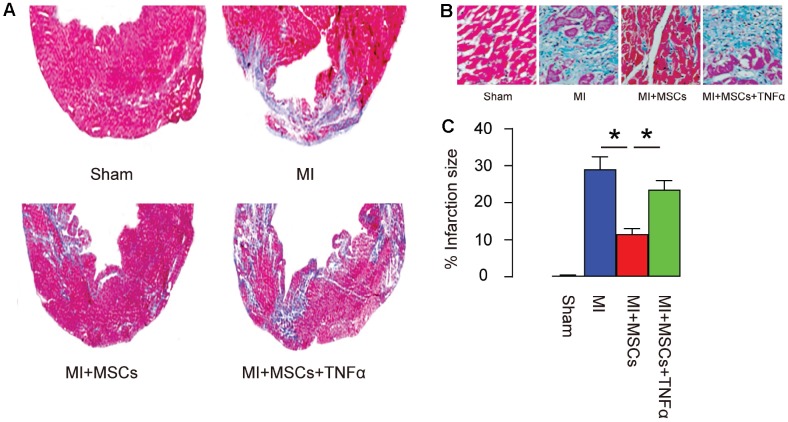
**MSC-induced M2-polarization of macrophages is essential for reducing infarction area in MI-mice.** (**A**–**B**) Masson's trichrome staining to determine the fibrotic heart tissue, shown by gross images (**A**), by representative histological images (**B**), and by quantification (**C**). *p<0.05. N=5.

Next, we used Masson's trichrome staining to assess the fibrosis levels in these mice. We found that MI significantly increased the infarction area, which MSC transplantation significantly reduced MI-induced infarction area, shown by gross images (Figure 4A), and by quantification (Figure 4B). The reduction in MI-induced infarction area by MSC-transplantation was significantly attenuated by TNF-α, shown by representative images (Figure 4A–4B), and by quantification (Figure 4C). Thus, MSC-induced M2-polarization of macrophages is essential for reducing infarction area in MI-mice.

### Injection of TNF-α antagonizes MSC-induced M2-polarization of macrophages in MI-mice

**Figure 5:**
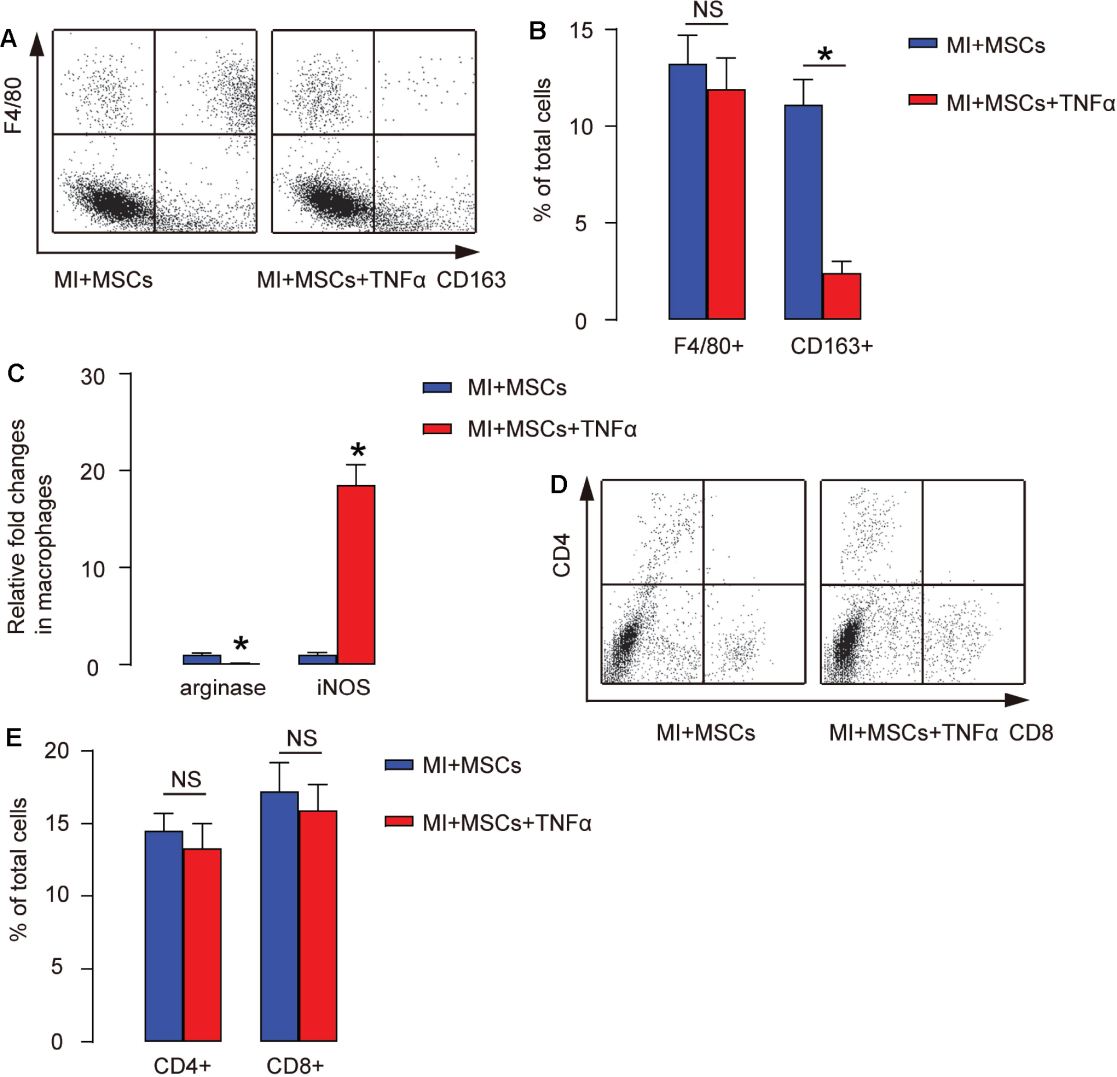
**Injection of TNF-α antagonizes MSC-induced M2-polarization of macrophages in MI-mice**. (**A**–**D**) The injured heart at 4 weeks after transplantation was digested, and the dissociated cells were analyzed by F4/80 and CD163, shown by representative flow charts (**A**), by quantification (**B**), and by ELISA for arginase and iNOS (**C**), or analyzed by CD4 and CD8, shown by representative flow charts (**D**), and by quantification (**E**). In panel A, Y-axis is F4/80 staining, and the X-axis is CD163 staining. In panel C, Y-axis is CD4 staining, and the X-axis is CD8 staining. *p<0.05. NS: non-significant. N=5.

To confirm that injection of TNF-α in MI-mice preserved heart function through antagonizing MSC-induced M2-polarization of macrophages in vivo, we digested the heart at 4 weeks after treatments and analyzed F4/80 and CD163 for macrophages as well as CD4 (a T-helper cell marker) and CD8 (a cytotoxic T cell marker) by FACS. We found that the total number of macrophages (by ratio of F4/80+ to the total cells) in the MI-heart did not alter by injection of TNF-α, shown by representative flow charts (Figure 5A), and by quantification (Figure 5B). However, the total number of M2 macrophages (by ratio of CD163+ to the total cells) in the MI-heart significantly decreased by injection of TNF-α, shown by representative flow charts (Figure 5A), by quantification (Figure 5B), and by ELISA for arginase and iNOS (Figure 5C). On the other hand, the number of CD4+ or CD8+ cells in the MI-heart was not altered by injection of TNF-α, shown by representative flow charts (Figure 5D), and by quantification (Figure 5E). Together, these data suggest that injection of TNF-α antagonizes MSC-induced M2-polarization of macrophages, but does not affect T-cells in MI-mice.

## DISCUSSION

The therapeutic benefits of MSCs could be partially stemmed from their modulation of inflammation response, since after MSCs treatment, the features and properties of macrophages in the injured heart was significantly changed, while the macrophage phenotypic changes are more likely appearing a M2-like alteration. Compared to M1, M2 macrophages have less pro-inflammatory potential, but produce and release many cytokines and growth factors to improve cell survival, proliferation, and to reduce cellular apoptosis [24]. Of note, proinflammatory cytokine TNFα is a cytokine primarily released by M1 macrophages, and also have a functionality of inducing M1 macrophage differentiation. Here, we successfully used TNFα to antagonize MSC-induced M2-polarization of macrophages. Moreover, since TNFα may also induce T-cell differentiation and affect T-cell subpopulation, we checked the changes in CD4+ versus CD8+ cells. As both CD4+ and CD8+ cells were not altered by TNFα, its main target in the current experimental model should be macrophages. We have also tried to use other M2-differentiation inhibitory cytokines, e.g. IFN-gamma, but the positive effects on T-cell differentiation precluded us from drawing conclusion and thus was not used here.

The crosstalk between MSCs and macrophages has been acknowledged in other regenerating models. For example, on therapeutic approaches on liver fibrosis and lower limb ischemia in mice [25, 26]. The immunomodulatory effects of MSCs have also been demonstrated, such as sepsis [27], renal artery stenosis [28] and myocardial infarction [29]. A previous study has shown that the MSCs that were injected into atherosclerosis-mice were preferentially trafficked to the plaque and likely migrated toward macrophages [30].

Among all immune cells in the injured heart, macrophages are the main source of cytokine production and produce proinflammatory cytokines as well as anti-inflammatory cytokines. Proinflammatory cytokines such as TNFα could reinforce the tissue and cell damage in MI. On the other hand, anti-inflammatory cytokines such as IL-10 should be able to suppress proinflammatory cytokines production, inhibit matrix metalloproteinases to favor heart function recovery and myocardial cell survival, since IL-10 also induces a polarization of macrophage to M2 type. Co-culture of MSCs with macrophages showed conversion of macrophages into M2 phenotype, resulting in the secretion of IL-10 and decreasing the production of TNFα [31]. Since blocking IL-10 receptor in macrophages induced higher NF-кB activation [32], the suppressing effects of IL-10 on inflammatory mediators might also be attributed to the inhibition of NF-кB activity [33]. This signaling pathway network may regulate the crosstalk between MSCs and macrophages.

To the best of our knowledge, our study is the first one to reveal a meaningful regulatory relationship between MSCs and macrophages on MI, which deserves further investigation on the detailed mechanisms that could provide important evidence for future application in treating MI in patients.

## MATERIALS AND METHODS

### Protocol approval

All the cell and animal experimental methods have received approval from the research committee at the Shanghai Chest Hospital.

### Manipulation of mouse MSCs

MSCs were obtained from euthanized male C57/BL6 mice of 12 weeks of age (Shanghai Laboratory Animal Center, Shanghai, China) and cultured in specific media as described before [16]. Phenotype analysis was determined by flow cytometry analysis and adipocyte, osteocyte and chondrocyte differentiation assay with corresponding kits (American Type Culture Collection (ATCC), Rockville, MD, USA; Catalog number: PCS-500-052, PCS-500-050 and PCS-500-051), and evaluated by Oil red O staining, Von kossa staining and Alcian blue staining, respectively.

### Flow cytometry

The flow cytometry for MSC surface markers included PEcy7-conjugated anti-Sca-1, CD105, CD90, CD45, CD34 and HLA-DR (Becton-Dickinson Biosciences, Shanghai, China). Macrophage or T-cell subtype analysis used PEcy5-conjugated anti-F4/80 or anti-CD4, and APC-conjugated anti-CD163 or anti-CD8 (Becton-Dickinson Biosciences). Flow cytometry data were analyzed and presented with FlowJo software (Flowjo LLC, Ashland, OR, USA).

### MI mouse model, MSC transplantation and injection of TNFα

MI was induced in male C57/BL6 mice at 12 weeks of age by ligation of the left anterior descending artery, as described [16]. One hour after ligation, mice received injection of saline (Sham or MI) or 6X105 MSCs at 6 points along the ligation. Recombinant mouse TNF-α (Ab9642, Abcam, Seattle, WA, USA) was dissolved in sterile saline prior to intraperitoneal injection twice per week at a dose of 200μg/kg. The mice were then kept for 4 weeks before analysis.

### Hemodynamic assessments

After anesthetization, ventricular catheterization was performed on mice, as described [16].

### ELISA and immunostaining

ELISA was done using appropriate kits. Heart tissue was fixed in 4% formalin, followed by paraffin embedding and sectioning. The immunostaining for F4/80 (Invitrogen, Shanghai, China) or CD86 (Abcam) was done as routine. Masson's trichrome staining was done with a specific kit (Sigma-Aldrich) as introduced by the manual.

### Statistical analysis

GraphPad prism version 8.0 (GraphPad Software, Inc. La Jolla, CA, USA) was used to analyze the data with a one-way analysis of variance (ANOVA) test followed by the Fisher’s Exact Test to compare two groups. All values represent the mean ± standard deviation (SD). A value of p<0.05 was considered statistically significant after Bonferroni correction.
